# Self-reported hearing difficulty in workers exposed to industrial dust in southern Brazil

**DOI:** 10.1590/2317-1782/20212020402

**Published:** 2021-10-18

**Authors:** Caroline Janaina de Jesus, Danúbia Hillesheim, Fernanda Zucki

**Affiliations:** 1 Departamento de Fonoaudiologia, Universidade Federal de Santa Catarina – UFSC - Florianópolis (SC), Brasil.

**Keywords:** Hearing, Occupational Exposure, Noise-Induced Hearing Loss, Dust, Occupational Health

## Abstract

**Purpose:**

To investigate the association between self-reported hearing difficulties and occupational exposure to industrial dust in workers in southern Brazil.

**Methods:**

This is a cross-sectional analytical study conducted with data from the National Health Survey (Pesquisa Nacional de Saúde, 2013). The dependent variable was self-reported hearing impairment, and the primary independent variable was occupational exposure to industrial dust. The covariables were: sex, skin color, age in complete years, exposure to noise, and exposure to chemical substances. The variables of occupational exposure to noise and chemical substances were used as adjustment variables (confounding variables), and the analyses were stratified per state (Santa Catarina, Paraná, and Rio Grande do Sul) to verify the difference in magnitude results per region. For the crude and adjusted analysis, the odds ratio (OR) was used as a measure of association, estimated through the logistic regression analysis. The data were analyzed through the software Stata, version 14.

**Results:**

Regarding the main exposure, 10.1% of the sample (n = 490) reported being exposed to industrial dust in an occupational environment, while 7.0% reported hearing impairment. In the final analysis, workers exposed to industrial dust were 1.77 times more likely to report hearing impairment when compared to individuals not exposed to this agent.

**Conclusion:**

There was an association between hearing impairment and exposure to industrial dust in workers in the southern region of Brazil.

## INTRODUCTION

Hearing is considered one of the senses that most influence the individual’s relationship with the environment, being fundamental in the acquisition and development of oral language and auditory skills ^(1)^. However, despite its importance, it is one of the most prevalent disabilities worldwide, being reported in over 5% of the world’s population (466 million people) and, in Brazil, according to data from the Brazilian Institute of Geography and Statistics (IBGE) of 2010, this problem affected about 5.1% of Brazilians, being considered the third most prevalent disability in the country ^(2-4)^.

Noise-induced hearing loss (NIHL) is commonly related to work conditions, and in Brazil, it is considered one of the leading health problems among workers ^(5)^. It is important to emphasize that occupational hearing loss (OHL) arises not only from exposure to high levels of sound pressure but also through exposure to ototoxic chemicals, vibrations, physical traumas, and dust ^(6,7)^.

Besides the hearing problems that arise as a result of the work, acquired hearing loss can bring numerous challenges for communication and interpersonal interaction and affect family relationships, self-esteem, and the efficiency in the execution of the auditory function ^(7,8)^.

Dust, little studied and cited in the literature, has been pointed out by some studies as a possible risk factor for the onset of hearing changes in the occupational environment ^(7-10)^. Dust is defined as a solid fragment that became powder or fine particles through mechanical disruption. The size and nature of the dust can indicate the degree of danger it represents to humans ^(11,12)^. The agricultural sector and civil construction are the main sectors that present dust as an integral agent of their activities, the first for involving work with cement and substances that make up the mortar, such as sand, clay and lime, and the last due to grain and animal confinement operations ^(7,8,13,14)^.

From the pathophysiology viewpoint, in the occupational activity, the dust can cause hearing loss due to ototoxic substances in its composition, which can cause cochlear dysfunction ^(7,8)^. Chemicals such as solvents, heavy metals, asphyxiants, and organophosphorus pesticides have been highlighted as potential ototoxic elements that are very present in occupational environments. However, each substance differs in its molecular structure and may act on different points of the auditory system ^(15)^.

The National Health Survey (Pesquisa Nacional de Saúde - PNS – 2013) was carried out in 2013 by the Ministry of Health in partnership with the Oswaldo Cruz Foundation and the Brazilian Institute of Geography and Statistics (IBGE). This research consists of a survey with household information, general characteristics of all residents of this household and the data of an adult resident who is 18 years of age or older. The general objective of the research is to produce data at the national level on the health situation, the lifestyle of the Brazilian population, and health care regarding access to and use of health services, preventive actions, continuity of care, and financing of care.

Given the above, as a representative population-based study, the National Health Survey (PNS) contributes much to the construction and assessment of public policies in the areas of promotion, surveillance and health care of the Unified Health System (SUS) and directing attention to groups and demands that require more priority ^(16,17)^.

Thus, the objective of this study is to investigate the association between self-reported hearing impairment and occupational exposure to industrial dust in workers in southern Brazil.

## METHODS

This article is the result of a cross-sectional and analytical study carried out with data from the survey of the National Health Survey (Pesquisa Nacional de Saúde – PNS – 2013), applied by IBGE technicians in 2013, with the participation of 205,546 adults (18 ≥ years) interviewed in 60,202 households. Our sample was composed of 4,875 adult workers (18 ≥ years) who were working within the reference week in the southern region of Brazil (Santa Catarina, Paraná, and Rio Grande do Sul). The selection of those participants was based on their positive answer to question E1 of Module E of the questionnaire: “In the week of July 21 to 27, 2013, did you work or intern for at least one hour in any cash-paid activity?” Detailed information on the sampling and data collection process has been described previously ^(18,19)^.

The dependent variable was self-reported hearing impairment, obtained through the question: *“In general, what is your degree of difficulty in hearing?*” The question presented the following categories of answers: none, mild, medium, intense, and unable. Those who reported having mild, medium, and severe hearing difficulty or being unable to hear were categorized as positive hearing impairment (yes), and individuals who reported having “none” were considered without hearing impairment (no).

The primary independent variable was occupational exposure to industrial dust, based on question M01108: *“Thinking about all your work, are you exposed to industrial dust (marble dust) that may affect your health?”* contained in Module M – Information for future contacts, work characteristics, and social support. The categories of answers covered were 1 (yes) or 2 (no). It is necessary to highlight that marble powder, contained in the question, consists only of a typical example of this occupational agent, an aspect observed in other questions of the PNS (2013), aiming at the contextualization of the question for the interviewee. In this sense, the interviewers were guided and properly instructed in the training process about this aspect, aiming at adequate data collection. The covariates were: sex (male; female), skin color (white; black; pardo; others), age in complete years (18 to 39; 40 to 59; 60 or more), noise exposure (no; yes) and exposure to chemicals (no; yes). The variables of occupational exposure to noise and chemicals were used as adjustment variables (confounding variables), as individuals could be exposed to these substances simultaneously.

Regarding the statistical analysis, initially, the data were represented by absolute and relative frequencies, with their respective 95% confidence intervals (95% CI). The analyses were stratified by the South region and by each state (Santa Catarina, Paraná, and Rio Grande do Sul), so we can verify the difference in the magnitude of the results by region.

Subsequently, Pearson’s chi-squared test was applied to test the difference in proportions of the categories of the variables analyzed. For the crude (bivariate) and the adjusted analyses, the odds ratio (OR) was used as a measure of association, estimated through logistic regression analysis, considering the design effect and the sample weights of the database in the analyses. The primary exposure variable was adjusted for all study covariates (sex, skin color, age, noise, chemicals) by the direct selection method, regardless of the p-value in the crude analysis. A statistically significant association was admitted when the probability of having occurred was equal to or less than 0.05, i.e., p ≤ 5%. Data were analyzed through the software Stata, version 14.

The PNS (National Health Survey) was approved by the National Research Ethics Commission (CONEP) – on July 8, 2013, under number 10853812.7.0000.0008 – of the National Health Council (Conselho Nacional de Saúde - CNS). All individuals who agreed to participate in the research signed an Informed Consent Form (ICF) ^(20)^.

## RESULTS

A total of 4,875 workers participated in this study, most of them male (52.2%), who reported being between 18 and 39 years of age (52.5%) and having white skin color (79.4%). Regarding the main exposure, 10.1% of the sample (n=490) answered that they were exposed to industrial dust in an occupational environment. Hearing loss was reported by 7.0% of the workers (Table 1).

**Table 1:** Description of the general characteristics of the sample. National Health Survey (Pesquisa Nacional de Saúde, 2013)

**Variable**	**Southern Region (n=4,875)**	
	n. (%)	CI 95%
**Sex**		
Male	2,547 (52.2)	50.8-53.6
Female	2,328 (47.7)	46.3-49.1
**Skin color**		
White	3,869 (79.4)	78.2-80.4
Black	238 (4.9)	4.3-5.5
Pardo	735 (15.0)	14.0-16.1
Others	33 (0.7)	0.4-0.9
**Age**		
18 to 39	2,552 (52.4)	50.9-53.7
40 to 59	1,947 (39.9)	38.7-41.3
60 or older	375 (7.7)	6.9-8.4
**Noise**		
No	3,161 (64.8)	63.4-66.1
Yes	1,714 (35.2)	33.8-36.5
**Chemical substances**		
No	3,795 (77.8)	76.6-78.9
Yes	1,080 (22.2)	21.0-23.3
**Industrial Dust**		
No	4,385 (89.9)	89.0-90.7
Yes	490 (10.1)	9.2-10.9
**Hearing Impairment**		
No	4,535 (93.0)	92.2-93.7
Yes	340 (7.0)	6.2-7.7

**Caption**: 95% CI = 95% confidence interval

Table 2 presents the characteristics of the sample according to the federative units of the South region. The highest prevalence of exposure to dust was recorded in the state of Santa Catarina (13.9%), followed by Paraná (9.2%) and Rio Grande do Sul (8.8%). The hearing difficulty was the same for the regions of Santa Catarina and Rio Grande do Sul (7.7%), and a lower proportion was observed in the region of Paraná (5.9%) (Table 2).

**Table 2:** Description of the characteristics of the sample according to states of the South region. National Health Survey (Pesquisa Nacional de Saúde, 2013)

**Variable**	**Paraná** *(n=1,974)*		**Santa Catarina** *(n=1,037)*		**Rio Grande do Sul** *(n=1,864)*	
	n. (%)	CI 95%	n. (%)	CI 95%	n. (%)	CI 95%
**Sex**						
Male	1,040 (52.7)	50.4-54.8	522 (50.3)	47.2-53.3	985 (52.8)	50.5-55.1
Female	934 (47.3)	45.1-49.5	515 (49.7)	46.6-52.7	879 (47.2)	44.8-49.4
**Skin color**						
White	1,391 (70.5)	68.4-72.4	897 (86.5)	84.2-88.4	1,581 (84.8)	83.1-86.3
Black	67 (3.4)	2.6-4.2	46 (4.5)	(3.3–5.8)	125 (6.7)	5.6-7.9
Pardo	499 (25.3)	23.4-27.2	87 (8.4)	6.8-10.2	149 (8.0)	6.8-9.3
Others	17 (0.9)	0.5-1.3	7 (0.7)	0.3-1.4	9 (0.5)	0.2-0.9
**Age**						
18 to 39	1,086 (55.0)	52.8-57.2	560 (54.0)	50.9-57.0	907 (48.7)	46.3-50.9
40 to 59	746 (37.8)	35.6-39.9	422 (40.7)	37.7-43.7	779 (41.8)	39.5-44.0
60 or older	142 (7.2)	6.1-8.4	55 (5.3)	4.0-6.8	178 (9.5)	8.2-10.9
**Noise**						
No	1,265 (64.0)	61.9-66.1	644 (61.1)	59.1-65.0	1,252 (67.2)	64.9-69.2
Yes	709 (36.0)	33.8-38.0	393 (37.9)	34.9-40.8	612 (32.8)	30.7-35.0
**Chemical substances**						
No	1,569 (79.5)	77.6-81.2	810 (78.1)	75.4-80.5	1,416 (76.0)	73.9-77.8
Yes	405 (20.5)	18.7-22.3	227 (21.9)	19.4-24.5	448 (24.0)	22.1-26.0
**Industrial Dust**						
No	1,792 (90.8)	89.4-91.9	893 (86.1)	83.8-88.0	1,700 (91.2)	89.8-92.4
Yes	182 (9.2)	8.0-10.5	144 (13.9)	11.9-16.1	164 (8.8)	7.5-10.1
**Hearing Impairment**						
No	1,858 (94.1)	92.9-95.0	957 (92.3)	90.4-93.7	1,720 (92.3)	90.9-93.4
Yes	116 (5.9)	4.9-7.0	80 (7.7)	6.2-9.5	144 (7.7)	6,5-9,0

**Caption**: 95% CI = 95% confidence interval

In the entire southern region, there was a higher prevalence of self-reported hearing difficulties in individuals exposed to industrial dust (12.2%) when compared to individuals not exposed to this agent, this difference being statistically significant (p<0.001) (Table 3).

**Table 3:** Prevalence of hearing impairment in individuals exposed to industrial dust in southern Brazil, crude and adjusted logistic regression analysis. National Health Survey (Pesquisa Nacional de Saúde, 2013)

**Industrial Dust**	**Hearing Impairment**					
	%	p*	Crude OR (95% CI)	p	Adjusted** OR (95% CI)	p
**Southern Region**		**<0.001**		**<0.001**		**0.001**
No	6.4		1.00		1.00	
Yes	12.2		2.04 (1.52-2.75)		1.77 (1.27-2.46)	
**Paraná**		**0.037**		**0.039**		0.123
No	5.5		1.00		1.00	
Yes	9.3		1.76 (1.02-3.01)		1.59 (0.88-2.88)	
**Santa Catarina**		**0.008**		**0.009**		0.179
No	6.8		1.00		1.00	
Yes	13.2		2.07 (1.19-3.58)		1.50 (0.83-2.70)	
**Rio Grande do Sul**		**0.001**		**0.001**		**0.006**
No	7.0		1.00		1.00	
Yes	14.6		2.25 (1.40-3.61)		2.15 (1.24-3.71)	

* Pearson's chi-squared test;

** Adjusted per sex, skin color, age, exposure to noise and exposure to chemicals

In the crude analysis, the data stratified by the federative units and the entire sample were associated with the study outcome. In the final adjusted analysis, workers in the southern region exposed to industrial dust were 1.77 times more likely to report hearing difficulties when compared to individuals not exposed to this agent (p=0.001). An association was also observed in Rio Grande do Sul (p=0.006), with a higher OR of 2.15 (Table 3).

## DISCUSSION

The present study showed that 10.1% of workers in the southern region were exposed to industrial dust in their occupational activity, while 7.0% reported hearing impairment. In the final analysis, workers exposed to industrial dust were 1.77 times more likely to report hearing difficulties when compared to individuals not exposed to this agent. Among the southern states of the country, workers from Rio Grande do Sul (p=0.006) had a higher odds ratio (OR=2.15) of reporting hearing loss, 2.15 times more likely than those not exposed to industrial dust.

The prevalence of self-reported hearing difficulties found in this study (7.0%) was slightly higher than that found in another population-based Brazilian study, with 6.8% of individuals reporting hearing difficulties ^(21)^. Although the previous study used the same database, it analyzed the total Brazilian sample, while the present study includes a smaller sample size, justifying the difference in the values found ^(21)^. In other studies, one on active urban mobility of adults with hearing loss and another on the prevalence of hearing loss in workers, there was the predominance of self-reported hearing loss of 17% and 2.7%, respectively ^(3,22)^.

In this study, the prevalence of exposure to industrial dust was 10.1% in workers in southern Brazil. On the other hand, two other studies, one in the agriculture sector and the other in the construction sector, found that 76% and 17.54% of the workers were exposed to dust ^(8,23)^.

According to the industry portal, the southern region of Brazil represents 17.8% of the country’s civil construction sector, especially Santa Catarina and Rio Grande do Sul^(24)^. Specifically, as the state of Rio Grande do Sul contributes the most in the civil construction sector of the region, with about 18.2% of participation, it is justifiable that it presents a higher odds ratio of hearing difficulty when workers are exposed to dust, since some studies state that the agricultural sector and civil construction are the main sectors that present dust as an integral agent of their activities ^(7,8,24)^.

Concerning the action of industrial dust on the auditory system, the literature explains in brief and few studies that this dust can cause hearing loss due to ototoxic or neurotoxic substances in its composition ^(7-10)^. Also, the combined exposure to industrial dust (lead dust, pesticides) and noise acts as a potentiator of the deleterious action in the cochlea and central auditory pathways, depending on the substance ^(25)^.

Considering that the individuals examined in this study could be synergistically exposed to other otoaggressive agents, the analyses were adjusted by the variables noise and chemicals present in the database. The combined exposure of those agents can induce changes and hearing loss since the effect is more significant than each agent’s action. This aspect was observed in a study that analyzed workers exposed to noise and styrene and found significantly worse hearing thresholds at 2, 3, 4 and 6 kHz when compared to workers exposed or not exposed to noise, demonstrating more important hearing loss when the exposure was combined ^(14,15)^.

In addition, those agents act differently on the auditory system and can be classified according to ototoxicity or neurotoxicity. While ototoxic substances affect inner ear structures, such as the hair cells existing in the cochlea and the neural pathways related to the peripheral auditory system (PAS), neurotoxic substances affect not only the PAS but also the central auditory nervous system (CANS), reaching the eighth cranial nerve and the central nervous system. This neurotoxic action can be seen by the losses, for example, in the auditory abilities, such as sound localization, speech discrimination in silence or in the presence of noise, even with normal auditory thresholds ^(26,27)^.

International and national studies have pointed out that there is an association between occupational exposure to dust and hearing problems. However, the literature still addresses few details about this agent and its performance in the auditory system ^(7-10)^. Thus, using the PNS data in this study, we can establish a regional overview of the workers’ hearing difficulties when exposed to industrial dust in their work.

Considering that the worker could present a hearing loss before occupational exposure and use a personal sound amplification product (PSAP), we found that only 11 individuals reported having used this equipment, not affecting the magnitude of the analyses.

The use of subjective, i.e., self-reported, answers can be considered a limitation of this study, as they may be underestimated or overestimated due to memory or self-perception bias ^(28)^. However, the literature indicates good sensitivity and specificity values for self-reported hearing loss in population surveys, presenting valid and reliable results ^(29,30)^. We must also highlight that there was no descriptive analysis of the professional activity of the sample. However, as this is a representative sample of the southern region of Brazil, we list and discuss the most prevalent professional activities in this region.

For future studies, we suggest collections with objective measures, such as the use of pure tone audiometry, to reduce the interference of subjective responses in the results. We also suggest investigating the association between hearing loss and specific ototoxic substances present in the dust to increase the knowledge of the professionals involved in health and safety at work. As this study is of an unprecedented character and exposure is still little studied and cited in the literature, we found it difficult to identify other research focused on the same theme.

## CONCLUSION

There was an association between self-reported hearing impairment and the exposure to industrial dust in workers in southern Brazil, chiefly in workers in Rio Grande do Sul. Given this finding, we suggest improvement in public policies on hearing health to develop more effective actions to prevent auditory diseases and monitor workers exposed to otoaggressive agents, such as industrial dust.
