# Supplementary material for: Self-reported hearing difficulty in workers exposed to industrial dust in southern Brazil
Source: Codas. 2021 Oct 18;34(1):e20200402. doi: 10.1590/2317-1782/20212020402 (PMC9886106; doi:10.1590/2317-1782/20212020402)
Supplement: Translation

# Translation: Dificuldade auditiva autorreferida em trabalhadores expostos à poeira industrial no sul do Brasil

**Authors:** Caroline Janaina de Jesus, Danúbia Hillesheim, Fernanda Zucki

**Affiliations:** 1 Departamento de Fonoaudiologia, Universidade Federal de Santa Catarina – UFSC - Florianópolis (SC), Brasil.

**Keywords:** Audição, Exposição Ocupacional, Perda Auditiva Induzida por Ruído, Poeira, Saúde do Trabalhador

## Abstract

**Objetivo:**

Investigar a associação entre dificuldade auditiva autorreferida e exposição ocupacional à poeira industrial em trabalhadores do sul do Brasil.

**Método:**

Trata-se de um estudo transversal analítico, realizado com dados do inquérito da Pesquisa Nacional de Saúde (2013). A variável dependente foi a dificuldade auditiva autorreferida e a variável independente principal foi a exposição ocupacional à poeira industrial. As covariáveis foram: sexo, cor da pele, idade em anos completos, exposição a ruído e exposição à substâncias químicas. As variáveis de exposição ocupacional a ruído e substâncias químicas foram utilizadas como variáveis de ajuste (variáveis de confusão) e as análises foram estratificadas por cada estado (Santa Catarina, Paraná e Rio Grande do Sul), com o intuito de verificar a diferença na magnitude dos resultados por região. Tanto para a análise bruta quanto para a ajustada, a razão de chance foi utilizada como medida de associação, estimada por meio da análise de regressão logística. Os dados foram analisados no software Stata, versão 14.

**Resultados:**

Com relação a exposição principal, 10,1% da amostra (n=490) afirmou estar exposta à poeira industrial em ambiente ocupacional, ao passo que 7,0% afirmou ter dificuldade auditiva. Na análise final, trabalhadores expostos a poeira industrial apresentaram 1,77 vezes mais chance de mencionar dificuldade auditiva, quando comparados a indivíduos não expostos a este agente.

**Conclusão:**

Houve associação entre dificuldade auditiva e exposição à poeira industrial em trabalhadores da região Sul do Brasil.

## INTRODUÇÃO

A audição é considerada um dos sentidos de maior influência na relação do indivíduo com o meio, sendo fundamental na aquisição e no desenvolvimento da linguagem oral e das habilidades auditivas ^(1)^. Entretanto, apesar de sua importância, é uma das deficiências com maior prevalência em todo o mundo, estando presente em mais de 5% da população mundial (466 milhões de pessoas) e, no Brasil, segundo dados do Instituto Brasileiro de Geografia e Estatística (IBGE) de 2010, esse problema afetava cerca de 5,1% dos brasileiros, sendo considerada a terceira deficiência mais prevalente no país ^(2-4)^.

A Perda Auditiva Induzida por Ruído (PAIR) está comumente relacionada ao trabalho, e, no Brasil, considera-se um dos principais problemas de saúde dos trabalhadores ^(5)^. É importante ressaltar que a Perda Auditiva Ocupacional (PAO) não surge apenas da exposição a elevados níveis de pressão sonora, mas também por meio da exposição a produtos químicos de natureza ototóxica, vibrações, traumas físicos e, ainda, pela poeira ^(6,7)^.

Além da preocupação com os problemas auditivos que surgem em decorrência do trabalho, uma perda auditiva adquirida pode trazer inúmeros desafios para a comunicação e o convívio interpessoal, pode ainda afetar as relações familiares, a autoestima e a eficiência da execução da função auditiva ^(7,8)^.

A poeira, pouco estudada e citada pela literatura, tem sido apontada por alguns estudos como possível fator de risco para o desencadeamento de alterações auditivas no ambiente ocupacional ^(7-10)^. Entende-se por poeira o fragmento sólido que se tornou pó ou partícula fina por meio de rompimento mecânico. O tamanho e a natureza de uma poeira podem apontar o grau de perigo que ela acarreta ao ser humano ^(11,12)^. O setor agropecuário e a construção civil constituem-se como os principais setores que apresentam a poeira como um agente integrante de suas atividades, o primeiro por envolver trabalho com cimento e de substâncias que compõem a argamassa, como areia, saibro e cal, e o último devido às operações de confinamento de grãos e animais ^(7,8,13,14)^.

Do ponto de vista da fisiopatologia, a poeira relacionada com a atividade ocupacional pode causar uma perda auditiva pelo envolvimento de substâncias ototóxicas em sua composição, sendo capaz de originar uma disfunção coclear ^(7,8)^. Produtos químicos como solventes, metais pesados, asfixiantes e pesticidas organofosforados, vêm se destacando como potenciais ototóxicos por estarem muito presentes nos ambientes ocupacionais, contudo, cada substância difere em sua estrutura molecular, podendo atuar sobre diferentes pontos do sistema auditivo ^(15)^.

A Pesquisa Nacional de Saúde (PNS – 2013) foi realizada em 2013 pelo Ministério da Saúde (MS), em parceria com a Fundação Oswaldo Cruz e o Instituto Brasileiro de Geografia e Estatística (IBGE). Essa pesquisa consiste em um inquérito com informações do domicílio, características gerais de todos os moradores desse domicílio e com os dados de um morador adulto, que tenha 18 anos de idade ou mais. O objetivo geral da pesquisa é produzir dados em âmbito nacional sobre a situação de saúde, o estilo de vida da população brasileira e sobre a atenção à saúde, no que se refere ao acesso e ao uso dos serviços de saúde, às ações preventivas, à continuidade dos cuidados e ao financiamento da assistência.

Diante do exposto, a Pesquisa Nacional de Saúde, por se tratar de um estudo de base populacional representativo, contribui demasiadamente na construção e na avaliação de políticas públicas nas áreas de promoção, vigilância e atenção à saúde do Sistema Único de Saúde (SUS), assim como direciona atenção aos grupos e demandas que requerem mais prioridade ^(16,17)^.

Dessa forma, o objetivo deste estudo é investigar a associação entre dificuldade auditiva autorreferida e exposição ocupacional à poeira industrial em trabalhadores do sul do Brasil.

## MÉTODO

Trata-se de um estudo transversal e analítico, realizado com dados do inquérito da Pesquisa Nacional de Saúde (PNS – 2013), que foi aplicado por técnicos do IBGE em 2013 e contou com a participação de 205.546 adultos (18 ≥ anos) entrevistados em 60.202 domicílios. A amostra deste estudo foi composta de trabalhadores adultos (18 ≥ anos) que estavam trabalhando dentro da semana de referência na região Sul do Brasil (Santa Catarina, Paraná e Rio Grande do Sul), ou seja, 4.875 trabalhadores. Esses participantes foram selecionados por responderam positivamente à questão E1 do Módulo E do questionário: *"Na semana de 21 a 27 de julho de 2013, você trabalhou ou estagiou, durante pelo menos uma hora, em alguma atividade remunerada em dinheiro?*". Informações minuciosas sobre o processo de amostragem e coleta de dados foram descritas anteriormente ^(18,19)^.

A variável dependente foi a dificuldade auditiva autorreferida, obtida por meio da pergunta: *“Em geral, que grau de dificuldade o(a) Sr(a) tem para ouvir?*”. A pergunta apresentava as seguintes categorias de resposta: nenhum, leve, médio, intenso e não consegue. As categorias leve, médio, intenso e não consegue foram categorizadas como dificuldade positiva para ouvir (sim) e os indivíduos que relataram “nenhum” foram considerados sem dificuldade auditiva (não).

A variável independente principal foi a exposição ocupacional à poeira industrial, a partir da pergunta M01108: *“Pensando em todos os seus trabalhos, o(a) Sr(a) está em exposição a poeira industrial (pó de mármore) que podem afetar a sua saúde?”* contida no Módulo M – Informações para futuros contatos, características do trabalho e apoio social. As categorias de resposta contempladas foram 1 (sim) ou 2 (não). Faz-se necessário destacar que, pó de mármore, contido na pergunta, consiste apenas em um exemplo comum desse agente ocupacional, aspecto observado em outras perguntas da PNS (2013), visando a contextualização da pergunta para o entrevistado. Nesse sentido, os entrevistadores foram orientados e devidamente instruídos no processo de capacitação acerca desse aspecto, visando a adequada realização da coleta de dados. As covariáveis foram: sexo (masculino; feminino), cor da pele (branca; preta; parda; outras), idade em anos completos (18 a 39; 40 a 59; 60 ou mais), exposição a ruído (não; sim) e exposição a substâncias químicas (não; sim). As variáveis de exposição ocupacional a ruído e a substâncias químicas foram utilizadas como variáveis de ajuste (variáveis de confusão), pois os indivíduos poderiam estar expostos simultaneamente a essas substâncias.

Com relação à análise estatística, inicialmente os dados foram representados por frequências absolutas e relativas, com seus respectivos Intervalos de Confiança de 95% (IC95%). As análises foram estratificadas pela região Sul e por cada estado (Santa Catarina, Paraná e Rio Grande do Sul), com o intuito de verificar a diferença na magnitude dos resultados por região.

Posteriormente, aplicou-se o teste de hipóteses de qui-quadrado de Pearson para testar a diferença de proporções das categorias das variáveis analisadas. Tanto para a análise bruta (bivariada) quanto para a ajustada, a *odds ratio* (OR) (Razão de Chances) foi utilizada como medida de associação, estimada por meio da análise de Regressão Logística, considerando-se, nas análises, o efeito de delineamento e os pesos amostrais do banco de dados. A variável de exposição principal foi ajustada por todas as covariáveis do estudo (sexo, cor da pele, idade, ruído, substâncias químicas) pelo método direto de seleção, independentemente do valor de p na análise bruta. Foi admitida associação estatisticamente significante quando a probabilidade de ter ocorrido foi igual ou menor do que 0,05, ou seja, p ≤ 5%. Os dados foram analisados no *software* Stata, versão 14.

A PNS foi aprovada pela Comissão Nacional de Ética em Pesquisa (CONEP) – em 8 de julho de 2013, sob o número 10853812.7.0000.0008 – do Conselho Nacional de Saúde (CNS). Todos os indivíduos que aceitaram participar da pesquisa assinaram um Termo de Consentimento Livre e Esclarecido (TCLE) ^(20)^.

## RESULTADOS

Participaram deste estudo 4.875 trabalhadores, a maioria do sexo masculino (52,2%), que referiu possuir entre 18 e 39 anos de idade (52,5%) e apresentar cor da pele branca (79,4%). Com relação à exposição principal, 10,1% da amostra (n=490) respondeu que estava exposta à poeira industrial em ambiente ocupacional. A dificuldade auditiva foi referida por 7,0% dos trabalhadores (Tabela 1).

**Tabela 1:** Descrição das características gerais da amostra. Pesquisa Nacional de Saúde (2013)

**Variável**	**Região Sul** *(n=4.875)*	
	n. (%)	IC 95%
**Sexo**		
Masculino	2.547 (52,2)	50,8-53,6
Feminino	2.328 (47,7)	46,3-49,1
**Cor da pele**		
Branca	3.869 (79,4)	78,2-80,4
Preta	238 (4,9)	4,3-5,5
Parda	735 (15,0)	14,0-16,1
Outras	33 (0,7)	0,4-0,9
**Idade**		
18 a 39	2.552 (52,4)	50,9-53,7
40 a 59	1.947 (39,9)	38,7-41,3
60 ou mais	375 (7,7)	6,9-8,4
**Ruído**		
Não	3.161 (64,8)	63,4-66,1
Sim	1.714 (35,2)	33,8-36,5
**Substâncias químicas**		
Não	3.795 (77,8)	76,6-78,9
Sim	1.080 (22,2)	21,0-23,3
**Poeira industrial**		
Não	4.385 (89,9)	89,0-90,7
Sim	490 (10,1)	9,2-10,9
**Dificuldade auditiva**		
Não	4.535 (93,0)	92,2-93,7
Sim	340 (7,0)	6,2-7,7

**Legenda**: IC95% = intervalo de 95% de confiança

Na Tabela 2 são apresentadas as características da amostra de acordo com as Unidades Federativas da região Sul. Maior prevalência de exposição à poeira foi registrada no estado de Santa Catarina (13,9%), seguido pelo Paraná (9,2%) e Rio Grande do Sul (8,8%). A dificuldade auditiva foi a mesma para as regiões de Santa Catarina e Rio Grande do Sul (7,7%), e menor proporção foi observada na região do Paraná (5,9%) (Tabela 2).

**Tabela 2:** Descrição das características da amostra segundo estados da região Sul. Pesquisa Nacional de Saúde (2013).

**Variável**	**Paraná** *(n=1.974)*		**Santa Catarina** *(n=1.037)*		**Rio Grande do Sul** *(n=1.864)*	
	n. (%)	IC 95%	n. (%)	IC 95%	n. (%)	IC 95%
**Sexo**						
Masculino	1.040 (52,7)	50,4-54,8	522 (50,3)	47,2-53,3	985 (52,8)	50,5-55,1
Feminino	934 (47,3)	45,1-49,5	515 (49,7)	46,6-52,7	879 (47,2)	44,8-49,4
**Cor da pele**						
Branca	1.391 (70,5)	68,4-72,4	897 (86,5)	84,2-88,4	1.581 (84,8)	83,1-86,3
Preta	67 (3,4)	2,6-4,2	46 (4,5)	3,3-5,8	125 (6,7)	5,6-7,9
Parda	499 (25,3)	23,4-27,2	87 (8,4)	6,8-10,2	149 (8,0)	6,8-9,3
Outras	17 (0,9)	0,5-1,3	7 (0,7)	0,3-1,4	9 (0,5)	0,2-0,9
**Idade**						
18 a 39	1.086 (55,0)	52,8-57,2	560 (54,0)	50,9-57,0	907 (48,7)	46,3-50,9
40 a 59	746 (37,8)	35,6-39,9	422 (40,7)	37,7-43,7	779 (41,8)	39,5-44,0
60 ou mais	142 (7,2)	6,1-8,4	55 (5,3)	4,0-6,8	178 (9,5)	8,2-10,9
**Ruído**						
Não	1.265 (64,0)	61,9-66,1	644 (61,1)	59,1-65,0	1.252 (67,2)	64,9-69,2
Sim	709 (36,0)	33,8-38,0	393 (37,9)	34,9-40,8	612 (32,8)	30,7-35,0
**Substâncias químicas**						
Não	1.569 (79,5)	77,6-81,2	810 (78,1)	75,4-80,5	1.416 (76,0)	73,9-77,8
Sim	405 (20,5)	18,7-22,3	227 (21,9)	19,4-24,5	448 (24,0)	22,1-26,0
**Poeira industrial**						
Não	1.792 (90,8)	89,4-91,9	893 (86,1)	83,8-88,0	1.700 (91,2)	89,8-92,4
Sim	182 (9,2)	8,0-10,5	144 (13,9)	11,9-16,1	164 (8,8)	7,5-10,1
**Dificuldade auditiva**						
Não	1.858 (94,1)	92,9-95,0	957 (92,3)	90,4-93,7	1.720 (92,3)	90,9-93,4
Sim	116 (5,9)	4,9-7,0	80 (7,7)	6,2-9,5	144 (7,7)	6,5-9,0

**Legenda**: IC95% = intervalo de 95% de confiança

Em toda a região Sul, houve maior prevalência de dificuldade auditiva autorreferida em indivíduos expostos à poeira industrial (12,2%), quando comparados a indivíduos não expostos a esse agente, sendo essa diferença estatisticamente significante (p<0,001) (Tabela 3).

**Tabela 3:** Prevalência de dificuldade auditiva em indivíduos expostos à poeira industrial na região sul do Brasil, análise de regressão logística bruta e ajustada. Pesquisa Nacional de Saúde (2013)

**Poeira industrial**	**Dificuldade auditiva**					
	%	p*	OR bruta (IC95%)	p	OR ajustada** (IC95%)	p
**Região Sul**		**<0,001**		**<0,001**		**0,001**
Não	6,4		1,00		1,00	
Sim	12,2		2,04 (1,52-2,75)		1,77 (1,27-2,46)	
**Paraná**		**0,037**		**0,039**		0,123
Não	5,5		1,00		1,00	
Sim	9,3		1,76 (1,02-3,01)		1,59 (0,88-2,88)	
**Santa Catarina**		**0,008**		**0,009**		0,179
Não	6,8		1,00		1,00	
Sim	13,2		2,07 (1,19-3,58)		1,50 (0,83-2,70)	
**Rio Grande do Sul**		**0,001**		**0,001**		**0,006**
Não	7,0		1,00		1,00	
Sim	14,6		2,25 (1,40-3,61)		2,15 (1,24-3,71)	

* Teste qui-quadrado de Pearson;

** Ajustado por sexo, cor da pele, idade, exposição a ruído e exposição a substâncias químicas

Na análise bruta, os dados estratificados por Unidades Federativas e toda a amostra apresentaram associação com o desfecho do estudo. Na análise final ajustada, trabalhadores da região sul expostos à poeira industrial apresentaram 1,77 vezes mais chance de referir dificuldade auditiva, quando comparados a indivíduos não expostos a esse agente (p=0,001). Também se observou associação no Rio Grande do Sul (p=0,006), com uma razão de chances maior (OR), igual a 2,15 (Tabela 3).

## DISCUSSÃO

O presente estudo apontou que 10,1% dos trabalhadores da região sul estavam expostos à poeira industrial em sua atividade ocupacional, ao passo que 7,0% referiram dificuldade auditiva. Na análise final, trabalhadores expostos à poeira industrial apresentaram 1,77 vezes mais chances de referir dificuldade auditiva, quando comparados a indivíduos não expostos a esse agente. Entre os estados do sul do país, os trabalhadores do Rio Grande do Sul (p=0,006) apresentaram uma razão de chances maior (OR=2,15) de referir dificuldade auditiva, tendo 2,15 vezes mais chances se comparados aos não expostos.

A prevalência de dificuldade auditiva autorreferida encontrada neste estudo (7,0%) foi ligeiramente maior que a encontrada em outro estudo brasileiro de base populacional, com 6,8% dos indivíduos relatando dificuldade para ouvir ^(21)^. Apesar de o estudo anterior utilizar a mesma base de dados, nele analisou-se a amostra total brasileira, enquanto a presente pesquisa contempla um recorte menor da amostra, justificando a diferença dos valores encontrados ^(21)^. Já em outros estudos, um sobre mobilidade urbana ativa de adultos com perda auditiva e outro sobre a prevalência de perda auditiva em trabalhadores, foi encontrada uma prevalência de perda auditiva autorreferida de 17% e 2,7%, respectivamente ^(3,22)^.

No presente estudo, a prevalência de exposição à poeira industrial foi de 10,1% nos trabalhadores na região do sul do Brasil. Em contrapartida, outros dois estudos realizados, um deles no setor da agricultura e o outro da construção civil, verificaram que 76% e 17,54% dos trabalhadores estavam expostos à poeira, respectivamente ^(8,23)^.

De acordo com o Portal da Indústria, a região do sul do Brasil representa 17,8% do setor da construção civil do país, com destaque para Santa Catarina e Rio Grande do Sul^(24)^. Especificamente, o Rio Grande do Sul é o estado que mais contribui no setor da construção civil da região, com cerca de 18,2% de participação, sendo justificável, portanto, que apresente uma maior razão de chances de dificuldade auditiva autorreferida quando os trabalhadores são expostos à poeira, já que alguns estudos afirmam que o setor agropecuário e a construção civil constituem-se como os principais setores que utilizam a poeira como um agente integrante de suas atividades ^(7,8,24)^.

Com relação à ação da poeira industrial no sistema auditivo, a literatura explica, de maneira breve e em poucos estudos, que essa poeira pode causar perda auditiva pela presença de substâncias ototóxicas ou neurotóxicas em sua composição ^(7-10)^. Além disso, a exposição combinada da poeira industrial (pó de chumbo, pesticidas) ao ruído age como potencializador da ação deletéria na cóclea e nas vias auditivas centrais, dependendo da substância ^(25)^.

Considerando que os indivíduos avaliados neste estudo poderiam estar expostos sinergicamente a outros agentes otoagressores, as análises foram ajustadas pelas variáveis ruído e substâncias químicas presentes no banco de dados. A exposição combinada desses agentes pode induzir a alterações e a perdas auditivas, já que o efeito é maior se comparado à ação de cada agente isolado. Esse aspecto foi observado em uma pesquisa que analisou trabalhadores expostos a ruído e a estireno e encontrou limiares auditivos significativamente piores em 2, 3, 4 e 6 kHz, quando comparados com trabalhadores expostos ou não expostos a ruído, demonstrando maior perda auditiva quando a exposição foi combinada ^(14,15)^.

Além disso, as características desses agentes diferem sua ação no sistema auditivo, podendo ser classificados quanto a sua ototoxicidade ou neurotoxicidade. Enquanto substâncias ototóxicas atingem estruturas de orelha interna, como as células ciliadas presentes na cóclea e nas vias neurais relacionadas ao Sistema Auditivo Periférico (SAP), as neurotóxicas afetam, além do SAP, o Sistema Nervoso Auditivo Central (SNAC), alcançando o oitavo nervo craniano e o sistema nervoso central. Essa ação de caráter neurotóxico pode ser vista pelos prejuízos, por exemplo, nas habilidades auditivas, como localização de som, discriminação de fala no silêncio ou na presença de ruído, mesmo com limiares auditivos normais ^(26,27)^.

Estudos internacionais e nacionais apontaram que existe associação entre exposição ocupacional à poeira e problemas auditivos, entretanto, a literatura ainda aborda poucos detalhes a respeito desse agente e de sua atuação no sistema auditivo ^(7-10)^. Dessa forma, utilizando os dados da PNS neste estudo, será possível estabelecer um panorama regional da dificuldade auditiva dos trabalhadores quando estes estão expostos à poeira industrial no exercício de sua ocupação.

Destaca-se ainda que, considerando que o trabalhador poderia apresentar uma perda auditiva prévia à exposição ocupacional e utilizar Aparelho de Amplificação Sonora Individual (AASI), constatou-se que apenas 11 indivíduos referiram fazer uso desse dispositivo, não afetando a magnitude das análises encontradas.

A utilização de respostas subjetivas, ou seja, autorreferidas, pode ser considerada uma limitação deste estudo, à medida que podem estar subestimadas ou superestimadas, devido ao viés de memória ou de autopercepção ^(28)^. Contudo, a literatura aponta bons valores de sensibilidade e especificidade para a perda auditiva autorreferida em inquéritos populacionais, apresentando resultados válidos e confiáveis ^(29,30)^. Faz-se necessário destacar ainda, que não houve uma análise descritiva da atividade profissional da amostra. Contudo, por tratar-se de uma amostra representativa da região sul do Brasil, foram elencadas e discutidas as atividades profissionais mais prevalentes nesta região.

Sugere-se, para estudos futuros, a realização de coletas com medidas objetivas, como a utilização da audiometria tonal liminar, a fim de reduzir a interferência de respostas subjetivas nos resultados. Sugere-se, ainda, a investigação da associação entre perda auditiva e substâncias ototóxicas específicas presentes na poeira, no intuito de ampliar o conhecimento de profissionais envolvidos na saúde e na segurança no trabalho, já que por este ser um estudo de caráter inédito e a exposição ainda ser pouco estudada e citada pela literatura, houve dificuldade para encontrar outros trabalhos envolvendo a mesma temática.

## CONCLUSÃO

Houve associação entre dificuldade auditiva autorreferida e exposição à poeira industrial em trabalhadores da região sul do Brasil, especialmente nos trabalhadores do Rio Grande do Sul. Diante de tal constatação, sugere-se aprimorar as políticas públicas em saúde auditiva com o propósito de desenvolver ações mais efetivas voltadas para a prevenção e o monitoramento auditivo dos trabalhadores expostos a agentes otoagressores, como a poeira industrial.
